# The effect of education around ethical principles on nurses’ perception to patient safety culture in an Iranian mental health inpatient unit: a pilot study

**DOI:** 10.1186/s12912-020-0402-7

**Published:** 2020-02-05

**Authors:** Behzad Razzani, Foroozan Atashzadeh-Shoorideh, Tayebeh Jamshidi, Maasoumeh Barkhordari-Sharifabad, Zahra Lotfi, Victoria Skerrett

**Affiliations:** 1 Student Research Committee, School of Nursing and Midwifery, Shahid Beheshti University of Medical Sciences, Tehran, Iran; 2 Department of Psychiatric Nursing, School of Nursing & Midwifery, Shahid Beheshti University of Medical Sciences, Vali-Asr Avenue, Cross of Vali-Asr and Hashemi Rafsanjani Highway, Opposite to Rajaee Heart Hospital, Tehran, 1996835119 Iran; 3 Department of Nursing, School of Medical Science, Yazd Branch, Islamic Azad University, Yazd, Iran; 4 Department of Nursing, Royal Free Hospital, London, UK; 5 Mental Health Nursing, School of Nursing and Midwifery, Birmingham City University, Birmingham, UK

**Keywords:** Ethical principles, Codes of ethics, Safety culture, Psychiatry, Nurse

## Abstract

**Background & objectives:**

Patient safety is a crucial factor in the provision of quality healthcare and is therefore a global health concern. It is an area in which ethical concerns and high-quality clinical practice are inextricably linked. This study investigates the effect of education around ethical principles on nurses’ perception of patient safety in a psychiatric unit.

**Materials & methods:**

This pre- and post-test descriptive study was conducted in a mental health inpatient unit in a hospital in Tehran, capital of Iran, in 2018. A total of 33 nurses, selected by census sampling, participated in the study. Data was collected using a demographics questionnaire and Hospital Survey on Patient Safety Culture (HSOPSC), and was analyzed with SPSS21.

**Results:**

The mean score of patient safety was 116.85 ± 9.98 before the educational intervention, 143.58 ± 7.21 immediately after intervention, and 153.12 ± 9.47 1 month after intervention. The rate of error report by most participants over the past 12 months was 3–5 and 6–10 events before intervention, and 6–10 events immediately after and 1 month after intervention. Also, 42.4% of the participants assessed patient safety after intervention as very good and 36.4% assessed it as acceptable and very good 1 month after intervention whereas most of the participants (45.5%) assessed patient safety as acceptable before intervention.

**Conclusion:**

Education on ethical principles exerts a positive effect on nurses’ perception of patient safety culture. Thus, it is recommended as an effective method of promoting nurses’ perception of this variable. In this way, healthcare quality and enhanced patient safety can be achieved.

## Introduction

Patient safety refers to avoiding any inadvertent detrimental damage to patients during care-giving, and is one of the influential parameters in quality healthcare [1]. Diagnostic errors (delayed diagnosis, lack of diagnosis, and misdiagnosis), pharmaceutical errors (incorrect dose or type of drug), decubitus ulcer, and falls are all examples of patient safety issues [2]. Although patient safety forms the most significant aspect (dimension) of healthcare quality and is considered as a global health concern, various studies have indicated that a considerable number of clients sustain damage during healthcare provision [3].

The results of previous studies demonstrate that about 3–17% of hospitalized patients suffer from untoward sequelae of complications while 30–70% of these events are preventable [4]. In England, untoward events occur in 10% of patient hospitalizations [5]; moreover, 16.6% of hospital admissions in Australia leads to unwanted events [6]. The rate of incidence of untoward events in Spain is reported as about 18.63% with 70% of them being preventable [7]. The most recent estimations of nosocomial mortality related to medical errors report that this rate is 2.5 times greater than the 98,000 deaths reported by Institute of Medicine in 1999 [8]. There are no complete statistics on medical errors and adverse events in Iran. Iranian Ministry of Health and Medical Education reported that Millions dollars are expended yearly to compensate for medical errors occurring during healthcare provision to patients [9, 10].. These adverse events and instances of unsafe care-giving predispose patients to untoward injuries and damages in patients resulting in detrimental consequences such as permanent injury, increased length of hospital stay, increased treatment costs, and even increased mortality rate. The adverse events further affect the post-discharge quality of life of patients increasing the odds of patient rehospitalization [11, 12].

Mental Health inpatient units are no exception. Patients in these wards are sometimes injured during healthcare provision. The difference is that the issues that threaten patient safety in these units mostly include, attempting suicide, self-injury, other-injury, injury relating to escape from hospital, falling from the height, physical, verbal, and mental violence, physical and mental issues due to the use of a seclusion room, rapid tranquilisation and physical restraint, sexual threat or assault and pharmaceutical errors [13]. Among these, falling is the most common adverse event in psychiatric wards [14]. Given the vulnerability of patients with mental health problems, safety in mental health inpatient units demands a great attention. The hazards that threaten patient safety in these wards affect not only the patients themselves, but also other patients, staff, and the public leading to an increased rate of risks [15].

In order to prevent and minimize these adverse effects and to improve the quality of health care, it is essential that safety is accepted as an intrinsic part of the health care culture in the organization and that the creation of a positive safety culture is consistently reinforced [8, 16]. Safety culture is considered a key underlying factor shaping the behaviors, perceptions, attitudes, and commitments of clinicians. It can affect the process of providing care and the effectiveness of interventions [17]. Such a patient safety culture emphasizes the importance of accurate report writing, and the analysis of near misses and medical errors that often lead to adverse health events [3].Having the appropriate atmosphere in terms of patient safety culture in a unit reduces the rate of errors and their adverse effects on patients [18, 19]. Since nurses are responsible for direct patient care and have more contact with patients, they play a major role in patient safety [10, 20]. Therefore, identifying nurses’ perceptions of patient safety culture is essential for managers [20].

Even though patient safety has attracted the attention of healthcare managers over the last decades, the ethical aspects of patient safety have not been sufficiently clarified. Patient safety is an issue which is fundamentally ethical in nature [21]. At first glance, nurses’ performance and clinical activity may seem to be separate from ethics. However, ethics and clinical performance are not practically separable [22]; rather, the observation of codes of ethics is related to nursing performance in care-giving [23]. On the other hand, promotion of nurses’ awareness of ethical principles and issues can lead to improved patient safety and quality care [24]. The incidence of ethical problems in mental health inpatient units and among psychiatric nurses is more prevalent due to the complexities of giving care to people with mental health problems where patients may be particularly vulnerable, where they may be driven to harm themselves and where nurses may find themselves confronted with dangerous and aggressive behavior [25]. In fact, due to the intrinsic nature of mental disorders, nurses will be required to make decisions, which are in the best interests of the patient. In this, they are required to observe the highest ethical principles in their clinical performance [26].

Considering the importance of patient safety in healthcare provision, this study explored the effect of education around ethical principles on nurses’ perception of patient safety culture in psychiatric unit of a hospital in Tehran/Iran.

## Methods

### Research design

This pre- and post-test descriptive study was carried out in 2018. The research setting included mental health inpatient units in one educational hospital in Tehran, Iran. The study examined four units: Psychiatric Emergency, Male Inpatient Mental Health Unit, Female Inpatient Mental Health Unit and Child Mental Health Unit. The study population included nurses working in above mentioned mental health units. Sampling was carried out by census method from nurses who met the inclusion criteria. Thus, there were 33 nurses (7 nurses in the psychiatric emergency, 8 nurses in the Male Inpatient Mental Health Unit, 9 nurses in the Female Inpatient Mental Health Unit and 9 nurses in the Child Mental Health Unit).

The inclusion criteria were: having at least a bachelor’s degree in nursing, employment in mental health for at least 1 year, and having not been trained in nursing ethical principles before entering the study over the past 6 months. Recruited participants were excluded if they had more than one absence from the educational sessions.

Nurses’ perception of patient safety culture (POPSC) was investigated first through Hospital Survey on Patient Safety Culture (HSOPSC); then, the educational content on nursing ethical principles was delivered to nurses during 4 educational sessions of 45–60 min duration held once per week for 1 month as face-to-face sessions and group discussion. In the first session, while explaining the objectives of the study, the necessity of the participants’ participation and the general topics, the ethics and ethical principles were discussed. In the second session, the discussion on the topic of Nursing Standards (professional ethics) was discussed. The third session was devoted to the topics of nursing codes of ethics and patient rights. Finally, the fourth session was assigned to discussing on ethical decision-making. All educational sessions were held in a hospital conference room. Moreover, to allow participants to review the taught materials, pamphlets containing the covered content were distributed. These nurses again completed the HSOPSC immediately after the training, and 1 month after intervention, in order for the effects of the education on nursing ethical principles, to be compared to the results obtained before the intervention.

### Data collection tools

This study used two questionnaires:

1. Demographics questionnaire including 7 items on age, gender, marital status, educational level, work experience in nursing, work experience in the mental health inpatient unit, and work hours per week.
2. The Persian version of HSOPSC was used to measure patient safety culture. This questionnaire was developed by Agency for Health Care Research and Quality in 2004 and is one of the commonly-used instruments in many studies for assessing patient safety culture. In this 42-item 5-point Likert scale, 12 items assess patient safety culture. These aspects include group working within the work unit (POPSC1), managers actions and expectations for promotion of safety (POPSC2), the direct managers’ activities in relation to the promotion of safety in work units and hospitals (POPSC3), organizational learning and continuous improvement (POPSC4), nurses’ general perception of patient safety (POPSC5), feedback and communication regarding errors (POPSC6), open communication in the work unit and hospital (POPSC7), rate of reported errors, (POPSC8), teamwork in the hospital (POPSC9), number of nurses and their workload (POPSC10), transfer from one unit to another unit (POPSC11), and non-punitive response to errors (POPSC12). Also, there are two items at the end of the questionnaire asking about first, the total score given to patient safety by the respondents in their own unit and second, about the total number of reports on medical errors.

To score the instrument, each of the 12 items is counted as a percent positive response. A 5-point Likert scales of agreement (strongly disagree to strongly agree) or frequency (never to always) was used to score the responses. Each item’s percent positive score consists of the number of positive responses to that item divided by the total number of responses to that item. On the basis of the questionnaire guideline, aspects that obtain at least 50% of positive points are in the acceptable status. Aspects with positive responses of greater than 75% are defined as the “strong points”, and aspects with positive responses less than 50% are the “in need of improvement” aspects.

Also at the end of the questionnaire, there are two questions, which ask respondents to give a score for patient safety on their unit (excellent to very weak) and how many reporting errors have occurred over the past 12 months. The number of errors and events over the past year, and responses, are classified in a 6-part spectrum ranging from 0 to 20+ cases. These two additional measures fall between 0 and 100% positive [27].

The criterion in this study was the total points given by the participants to HSOPSC; the greater the points, the more safe the hospital is, regarding patient safety culture [28]. The internal consistency correlation coefficient of the instrument was 0.85.

### Data analysis

The gleaned data was analyzed with SPSS21 using Mean ± SD, repeated measures of ANOVA, and percentage to determine POPSC before and after intervention.

## Results

The mean age of nurses was 35.84 ± 6.56 years, the mean nursing experience was 11.45 ± 5.56 years, the mean work experience on the mental health unit was 6.21 ± 4.52 years, and the mean work hours per week was 51.53 ± 5.13 h. Most participants (81.82%) were female, married (78.79%), held a BS in nursing (87.88%), and had rotating work shift (81.82%) (Table 1).

**Table 1:** Sociodemographic characteristics of study participant

Variables	M (SD)	n (%)
Age	35.56 (6.56)	
Gender		
Female		27 (81.82)
Male		6 (18.18)
Marital Status		
Single		7 (21.21)
Married		26 (78.79)
Level of Education		
Bachelor degree		29 (87.88)
Master degree		3 (9.09)
PhD degree		1 (3.03)
Nursing Experience	11.45 (5.56)	
Work experience in psychiatric ward	6.21 (4.52)	
Work hours per week	51.53 (5.13)	
Work Shifts		
Morning		5 (15.15)
Evening		1 (3.03)
Night		0 (0)
Rotating		27 (81.82)

The findings of the study showed that the POPSC ranged between 50 and 75% before intervention that was acceptable. The greatest points obtained (72.73%) belonged to POPSC1 and the smallest points obtained (37.73%) pertained to POPSC11. Before the intervention, all aspects except for POPSC2, POPSC7, POPSC11, and POPSC12 were within an acceptable range. The mean score of patient safety was 68.37% (143.58 ± 7.21) immediately after intervention that increased by 12.73%. Immediately after intervention, the highest score pertained to POPSC1 (83.33%) whereas the lowest score belonged to POPSC12 (49.55%). The three aspects “POPSC1” (83.33%), “POPSC4” (78.79%), and “POPSC6” (75.76%) that had acquired points greater than 75% were rendered as the “strong points” of patient safety culture (PSC). Additionally, all aspects were in at an acceptable level in this stage as their points increased compared to before intervention. Regarding the results 1 month after educational intervention, the mean score of patient safety was 72.91% (153.12 ± 9.47) that showed an increase compared to before and immediately after educational intervention. In the follow up stage, the highest score was obtained by POPSC1 (88.03%) and the lowest score was obtained by POPSC11 (56.67%); this was similar to the results before intervention in this regard. Furthermore, in the follow up investigation, the four aspects POPSC1 (88.03%), POPSC4 (84.04%), POPSC6 (81.82%), and POPSC8 (76.36%) with points greater than 75% were rendered as “strong points” (Table 2).

**Table 2:** POPSC scores in participants’ perspective before, after, and 1 month after educational intervention (the follow up stage)

Dimensions of POPSC	Before		Immediately after		Follow up		ANOVA
	Mean (SD)	Average score based on 100	Mean (SD)	Average score based on 100	Mean (SD)	Average score based on 100	
POPSC1	14.55 (2.17)	72.73	16.67 (1.96)	83.33	17.61 (1.69)	88.03	*F* = 27.75 *P* < 0.000
POPSC2	9.33 (2.34)	46.67	11.64 (2.04)	58.18	13.09 (1.94)	65.45	*F* = 41.87 *P* < 0.000
POPSC3	8.73 (1.4)	58.18	10.52 (0.97)	70.10	10.76 (1.06)	71.72	*F* = 38.85 *P* < 0.000
POPSC4	9.85 (1.77)	65.66	11.82 (1.38)	78.79	21.61 (1.64)	84.04	*F* = 36.05 *P* < 0.000
POPSC5	11.61 (2.6)	58.03	13.7 (1.91)	68.48	14.24 (2.57)	71.21	*F* = 21.89 *P* < 0.000
POPSC6	9.06 (1.66)	60.40	11.36 (2.07)	75.76	12.27 (1.74)	81.82	*F* = 34.65 *P* < 0.000
POPSC7	7.42 (1.54)	49.49	9.45 (1.75)	63.03	9.61 (1.68)	64.04	*F* = 28.86 *P* < 0.000
POPSC8	8.09 (2.02)	53.94	10.67 (2.41)	71.11	11.45 (1.79)	76.36	*F* = 38.70 *P* < 0.000
POPSC9	11.67 91.27)	58.33	12.85 (1.5)	64.24	13.88 (1.34)	69.39	*F* = 29.47 *P* < 0.000
POPSC10	10.97 (1.38)	54.85	14.3 (1.88)	71.52	14.79 (1.63)	73.94	*F* = 57.46 *P* < 0.000
POPSC11	7.55 (2.25)	37.73	10.7 (2.87)	53.48	11.33 (2.04)2	56.67	*F* = 34.24 *P* < 0.000
POPSC12	8.03 (1.78)	40.15	9.91 (2.01)	49.55	11.48 (1.56)	57.42	*F* = 36.46 *P* < 0.000
Total	116.85 (9.98)	55.64	143.58 (7.21)	68.37	152.12 (9.74)	72.91	

Besides, our findings suggested that, before intervention, most participants (39.4%) stated that there have been “between 3 and 5 events” and “between 6 to 10 events” over the last 12 months. Nevertheless, most participants reported “between 6 to 10 events” over the past 12 months immediately after and 1 month after intervention (Table 3).

**Table 3:** Frequency of events by nurses over the past 12 months

Number of Events	Before	After	Follow up
	Frequency (%)	Frequency (%)	Frequency (%)
no events	0 (0)	0 (0)	1 (3)
1–2 events	2 (6.1)	6 (18.2)	1 (3)
3–5 events	13 (39.4)	9 (27.3)	11 (33.3)
6–10 events	12 (39.4)	11 (33.3)	14 (42.4)
11–20 events	5 (15.2)	6 (18.2)	6 (18.2)
21 or more events	1 (3)	1 (3)	0 (0)
Total	33 (100)	33 (100)	33 (100)

The results indicated that most respondents (42.4%) assessed patient safety as very good immediately after intervention. In addition, most of them (36.4%) assessed patient safety as acceptable and very good 1 month after intervention whereas the majority of them (45.5%) assessed it as acceptable before intervention (Table 4).

**Table 4:** Absolute and relative frequency distributions of patient safety scoring (percentage)

Scoring to patient safety	Before	After	Follow up
	Frequency (%)	Frequency (%)	Frequency (%)
Very weak	0 (0)	0 (0)	0 (0)
Weak	8 (24.2)	7 (21.2)	3 (9.1)
Acceptable	15 (45.5)	8 (24.2)	12 (36.4)
Very good	9 (27.3)	14 (42.4)	12 (36.4)
Excellent	1 (3)	4 (12.1)	6 (18.2)
Total	33 (100)	33 (100)	33 (100)

## Discussion

This study investigated the effect of education about ethical principles on nurses’ perception of patient safety culture (POPSC) in mental health inpatient units. The statistical findings of the study revealed that the mean score of patient safety in all components, measured after and 1 month after educational intervention (the follow up stage), increased compared to before intervention. Besides, most participants assessed patient safety as acceptable before intervention whereas they assessed it as very good immediately after and 1 month after educational intervention. The results of other studies are consistent with our findings [4, 29–32]. For example, the study conducted in Jordan aiming at assessing mental health nurses’ perception of patient safety culture in the field of psychiatry demonstrated that two-thirds of nurses assessed patient safety as very good or excellent [29]. Also, the results of the study by Ricklin et al. (2019) in Switzerland indicated that patient safety is held to be crucial in this center so that 78% of physicians and 60% of nurses assessed patient safety in the study as very good and excellent [30]. Yet, unlike our results, their findings suggested weak status of patient safety culture [33–35]. The difference may be attributed to different organizational culture, the management conditions governing different hospitals, differences in structures of the related questionnaires and their analysis methods, presence or absence of occupational health unit in all hospitals, etc. and also the efficacy and efficiency of educational interventions of the present study. Patient safety culture may be related to a punitive culture governing the mentioned hospitals, unjust distribution of nurses among wards, and lack of head nurses’ and managerial support for patient safety [36], so that if the staff dare to report errors without any fear of scolding or punishment, they are assured that their errors do not work against themselves; rather, they provide useful constructive opportunities for them [37, 38].

The results of the present study indicated that the highest points (72.73%) acquired before intervention belonged to POPSC1 and the lowest (37.73%) belonged to POPSC11. POPSC1 deals with reciprocal interpersonal respectfulness of the staff toward each other and the importance of their team work. The obtained results are consistent with some studies [39, 40], that highlight the importance of POPSC1 in promoting patient safety culture. Consequently, it may be concluded that team work ought to be performed appropriately. Regarding the significance of team work, some studies have suggested that problems of patient safety culture are due to deficiencies and shortcomings in work processes within the units; also, weak rapport among the healthcare personnel is one of the main causes of medical errors in medical field [41]. Indeed, teamwork is the key to solving many problems in healthcare systems. Fortunately, this was carefully observed in the present study and is rendered as one of the strong points of the study. Moreover, the results of the study by Azami Aqdas et al. reported that the highest score belonged to POPSC2 (67.4%) [4], whereas in Ricklin et al.’s study, the highest score of positive responses pertained to POPSC12; this is not consistent with our findings [30].

POPSC11 pertains to loss of important information of patient care during work shift changes or during patient transfer among different wards in hospital. In the present study, this component acquired the lowest score among other aspects and was classified as an “in need of improvement” aspect. Nonetheless, POPSC11 is of utmost significance for patient care. Lack of proper correct communication and/or failure of IT system or inaccurate information may lead to untoward sequalae and potential adverse events jeopardizing patient safety. All these may bring about serious consequences for patients [41]. Of course, in the study by Ricklin et al., the smallest score of positive responses belonged to POPSC8 [30]; this is not consistent with our findings.

Moreover, the rate of error-related events were reported to be fewer after educational intervention compared to before intervention; still, this rate increased 1 month after completion of intervention. Anyway, the results of most similar studies demonstrated a low rate of error report by nurses so that in the study by Granel et al., 66% of individuals reported no medical error over the past year [39]. The results of the study by Azimi Aqdas et al. showed that most nurses (52.7%) reported no medical errors over the past 12 months [4]. Besides, in the investigation by Mahrous, 40% of the participants reported no medical error over the past 12 months [32]. It appears that factors such as culture and organizational atmosphere governing hospitals, insufficient awareness of nurses about positive consequences of error reports and the related learned tips, their fear of punishment and scolding, inefficacy of reports and assumptions of individualistic nature of errors hinder prompt, accurate, and comprehensive report of errors [42]. Another study attributed lack of error reporting to fear of manager’s or colleague’s reaction, fear of being scolded or labelled as incompetent, and patient’s negative attitude [43].

Our findings suggested that education of ethical principles fostered all aspects of patient safety culture. Consistent with the findings of the present study, Valenti et al. (2014) asserted in their study that patient safety culture is related to nurses’ ethical performance since management of risks and complications creates an ethical obligation for protection of patients, healthcare providers, and the public through principle of patient beneficence, absence of profiteering against patients, and promotion of health [44]. The results of other studies also suggested that the total score of patient safety increased significantly after educational intervention [45–48]. Contrary to the results of the present study, in the study by Ricklin et al. no significant difference was observed in the results before and after educational intervention of sensitization [30], an issue that may be attributed to different educational interventions.

## Conclusion

In this study, education of ethical principles resulted in increased POPSC of nurses working in mental health units. Thus, education of ethical codes and principles of nursing may be recommended as an efficient and efficacious method of promoting patient safety and enhancing quality care. Hospital managers are encouraged to provide such educational programs as part of their in-service curriculum in nursing and midwifery schools.

## Data Availability

Sharing the data is not possible due to an agreement with the participants on the confidentiality of the data.

## Abbreviations

HSOPSC: Hospital survey on patient safety culture
POPSC: Perception of patient safety culture
POPSC1: Group working within the work unit
POPSC10: Number of nurses and their workload
POPSC11: Transfer from one unit to another unit
POPSC12: Non-punitive response to errors
POPSC2: Managers actions and expectations for promotion of safety
POPSC3: The direct managers’ activities in relation to the promotion of safety in work units and hospitals
POPSC4: Organizational learning and continuous improvement
POPSC5: Nurses’ general perception of patient safety
POPSC6: Feedback and communication regarding errors
POPSC7: Open communication in the work unit and hospital
POPSC8: Rate of reported errors
POPSC9: Teamwork in the hospital
